# Association of *CD274* (PD-L1) Copy Number Changes with Immune Checkpoint Inhibitor Clinical Benefit in Non-Squamous Non-Small Cell Lung Cancer

**DOI:** 10.1093/oncolo/oyac096

**Published:** 2022-05-22

**Authors:** Karthikeyan Murugesan, Dexter X Jin, Leah A Comment, David Fabrizio, Priti S Hegde, Julia A Elvin, Brian Alexander, Mia A Levy, Garrett M Frampton, Meagan Montesion, Sameek Roychowdhury, Razelle Kurzrock, Jeffrey S Ross, Lee A Albacker, Richard S P Huang

**Affiliations:** Foundation Medicine, Inc., Cambridge, MA, USA; Foundation Medicine, Inc., Cambridge, MA, USA; Foundation Medicine, Inc., Cambridge, MA, USA; Foundation Medicine, Inc., Cambridge, MA, USA; Foundation Medicine, Inc., Cambridge, MA, USA; Foundation Medicine, Inc., Cambridge, MA, USA; Foundation Medicine, Inc., Cambridge, MA, USA; Foundation Medicine, Inc., Cambridge, MA, USA; Foundation Medicine, Inc., Cambridge, MA, USA; Foundation Medicine, Inc., Cambridge, MA, USA; James Cancer Hospital, Ohio State University Comprehensive Cancer Center, Columbus, OH, USA; Division of Hematology and Oncology, Medical College of Wisconsin, Milwaukee, WI, USA; Foundation Medicine, Inc., Cambridge, MA, USA; Department of Pathology, State University of New York Upstate Medical University, Syracuse, NY, USA; Foundation Medicine, Inc., Cambridge, MA, USA; Foundation Medicine, Inc., Cambridge, MA, USA

**Keywords:** non-small cell lung cancer, immunotherapy, comprehensive genomic profiling, real world evidence, *CD274*

## Abstract

**Background:**

We sought to characterize response to immune checkpoint inhibitor (ICI) in non-squamous non-small cell lung cancer (NSCLC) across various *CD274* copy number gain and loss thresholds and identify an optimal cutoff.

**Materials and Methods:**

A de-identified nationwide (US) real-world clinico-genomic database was leveraged to study 621 non-squamous NSCLC patients treated with ICI. All patients received second-line ICI monotherapy and underwent comprehensive genomic profiling as part of routine clinical care. Overall survival (OS) from start of ICI, for *CD274* copy number gain and loss cohorts across varying copy number thresholds, were assessed.

**Results:**

Among the 621 patients, patients with a *CD274* CN greater than or equal to specimen ploidy +2 (*N* = 29) had a significantly higher median (m) OS when compared with the rest of the cohort (*N* = 592; 16.1 [8.9-37.3] vs 8.6 [7.1-10.9] months, hazard ratio (HR) = 0.6 [0.4-1.0], *P*-value = .05). Patients with a *CD274* copy number less than specimen ploidy (*N* = 299) trended toward a lower mOS when compared to the rest of the cohort (*N* = 322; 7.5 [5.9-11.3] vs 9.6 [7.9-12.8] months, HR = 0.9 [0.7-1.1], *P*-value = .3).

**Conclusion:**

This work shows that *CD274* copy number gains at varying thresholds predict different response to ICI blockade in non-squamous NSCLC. Considering these data, prospective clinical trials should further validate these findings, specifically in the context of PD-L1 IHC test results.

## Teaser

This article reports on the association of immune checkpoint inhibitor (ICI) response to therapy in patients with nonsquamous non-small cell lung cancer across various *CD274*copy number gain and loss thresholds and at various cutoffs.

**Implications for Practice**

In this study of 621 non-squamous patients with non-small cell lung cancer (NSCLC) from a de-identified nationwide clinico-genomic database, patients with a *CD274* copy number (CN) of at least specimen ploidy +2, +3, +4 and at most specimen ploidy −1, −2, −3 showed varying responses to immune checkpoint inhibitors (ICIs). A *CD274* CN gain threshold of at least specimen ploidy +2 identified patients with a higher median OS. This work suggests that *CD274* CN thresholds can influence response to ICI and *CD274* CN as a potential biomarker for ICI in non-squamous NSCLC.

## Introduction

Immune checkpoint inhibitors (ICIs) have been approved for use in multiple tumor types and subsequently incorporated into the National Comprehensive Cancer Network (NCCN) guidelines, influencing real-world clinical management of patients with cancer.^1^ Despite this, only an estimated 12.5% of eligible (based on PD-L1 positivity) patients are reported to respond to ICI,^2^ while frequent immune-related adverse events are observed in ICI treated patients.^3,4^ Hence, it is of the utmost importance to further develop both positive and negative predictive biomarkers for ICI response.

PD-L1 expression as detected by immunohistochemistry (IHC) has identified a subset of tumors more responsive to ICI^5^ and is an US Food and Drug Administration (FDA) approved companion diagnostic (CDx) in multiple tumor types^6^; however, PD-L1 IHC testing is complex and remains insufficient to consistently predict response to ICI.^3,7-9^ In addition, tumor mutational burden high (TMB-High defined as TMB greater than or equal to 10 mutations/Megabase [muts/Mb]) and microsatellite instability-high (MSI-H) solid tumor patients are also eligible to receive ICI based on 2 pan solid tumor approvals.^10,11^ However, the clinical outcomes of ICI treatments in these biomarker positive patients is varied.^9,12^ Recently, interest has emerged in the study and development of composite biomarkers that incorporate both tumor cell intrinsic and tumor microenvironment derived predictors of ICI response.^13^

Both *CD274* (gene encoding PD-L1) gains and losses have been discussed in clinical studies as positive and negative predictive biomarkers for ICI in various tumor types.^14-19^ Inoue et al^20^ showed that *CD274* amplified tumors (defined as ploidy times 2 as detected by Fluorescence in situ hybridization [FISH]) when compared with tumors with PD-L1 polysomy and PD-L1 disomy had better survival outcomes to nivolumab after progression on prior therapy, with the 1-year OS rate being 100% (*N* = 5), 46% (*N* = 27) and 57.6% (*N* = 162), respectively, in a cohort of 194 patients with NSCLC. Goodman et al^18^ identified 9 *CD274* amplified (using comprehensive genomic profiling (CGP) and at a cutoff of ploidy +4) solid-tumor patients treated with ICI and reported an ORR of 66.7% and a median progression-free survival of 15.2 months. However, different assays and *CD274* copy number cutoffs were used in these different studies. Huang et al recently studied over 240 000 patient specimens across multiple tumor types^21^ that underwent CGP and showed that *CD274* copy number gains (defined as *CD274* copy number of at least specimen ploidy +1) were more prevalent than *CD274* amplifications (defined as *CD274* copy number of at least specimen ploidy +4) and also correlated with increased PD-L1 expression. As previously shown^21^ among 30 396 lung adenocarcinomas, we reported the prevalence of *CD274* copy number gains defined as *CD274* copy number of at least specimen ploidy +1, specimen ploidy +2, specimen ploidy +3, and specimen ploidy +4 as 15%, 5.1%, 1.8%, and 0.9%, respectively.

Due to the variable prevalence rates of positivity at different *CD274* copy number cutoffs and given the varying responses based on different *CD274* copy number cutoffs in the aforementioned clinical studies, it is imperative to find an optimal standardized copy number cutoff for *CD274* that is correlated with patient response to ICI in specific tumor types. Here, we investigate the association of ICI response with *CD274* copy number gains and losses at various cutoffs in a clinico-genomic cohort of 621 non-squamous patients with NSCLC.

## Materials and Methods

### Patients

This study used the nationwide (US-based) de-identified Flatiron Health-Foundation Medicine clinico-genomic database (CGDB). The de-identified data originated from approximately 280 cancer clinics (~800 sites of care). Retrospective longitudinal clinical data were derived from electronic health record data, comprising patient-level structured and unstructured data, curated via technology-enabled abstraction, and were linked to genomic data derived from FMI CGP tests in the CGDB by de-identified, deterministic matching.^22^ Institutional Review Board approval of the study protocol was obtained prior to study conduct and included a waiver of informed consent.

This study included 621 patients satisfying the following cohort inclusion criteria: (1) chart-confirmed diagnosis of non-squamous NSCLC (data collected through December 31, 2020), (2) Had at least 2 documented clinical visits in the Flatiron Health network on or after January 1, 2011, (3) Underwent CGP testing on a pathologist-confirmed non-squamous NSCLC tumor specimen, at FMI, on or after date of chart-confirmed initial diagnosis of non-squamous NSCLC, on a sample collected no earlier than 30 days before the Flatiron Health diagnosis date. (4) Wild-type for any oncogenic *EGFR* and *ALK* genomic alteration as determined by the FoundationOne and FoundationOne CDx CGP test (5) Were treated with second-line ICI monotherapy, specifically, Atezolizumab, Durvalumab, Nivolumab, or Pembrolizumab (Patients who had already received any form of ICI in the first-line setting were excluded). Patients were observed to have received a second-line ICI monotherapy between May 2015 and November 2020.

### Comprehensive Genomic Profiling

Clinical cases of non-squamous NSCLC (as diagnosed by the treating physician and confirmed on hematoxylin and eosin-stained slides) underwent CGP performed using the FoundationOne and FoundationOne CDx assays as described previously, in a Clinical Laboratory Improvement Amendments (CLIA) certified and College of American Pathologists (CAP) accredited laboratory.^23,24^ All samples submitted for sequencing featured a minimum of 20% tumor cell nuclear area and yielded a minimum of 50 ng of extracted DNA. CGP was performed on hybridization-captured, adapter-ligation based libraries, to identify genomic alterations (base substitutions, small insertions/deletions, copy number alterations and rearrangements) in greater than 300 cancer-associated genes, tumor mutational burden (TMB)^25^ and MSI.^26^

### *CD274* Copy Number Calling

Copy number alterations were detected using a comparative genomic hybridization-like method applied to next generation sequencing data.^23,27^ In the laboratory, each specimen was analyzed alongside a process-matched normal control (an internally validated mixture of 10 heterozygous diploid samples from the HapMap project), with custom algorithms to normalize the sequence coverage distribution across captured DNA regions. Log-ratios of normalized coverage data for exonic, intronic, and SNP targets accounting for stromal admixture, as well as genome-wide SNP frequencies, were used to generate the profiles. Using circular binary segmentation, custom algorithms further clustered groups of targets and SNP frequencies to define upper and lower bounds of genomic segments. Empirical Bayesian algorithms used a distribution of parameters including purity and base ploidy and probability matrices were derived using different statistical sampling methodologies to fit these data. Specimen-level ploidy was estimated as described by Sun et al^27^ Computational models were reviewed by expert analysts for each sample.^23^

### PD-L1 Expression

PD-L1 IHC testing was run and interpreted by experienced board-certified pathologists according to the manufacturer instructions in a CLIA-certified and CAP-accredited laboratory (Foundation Medicine, Inc, Morrisville, NC) for a subset of specimens in this CGDB cohort. DAKO PD-L1 IHC 22C3 pharmDx’s tumor proportion scoring (TPS) method was used to score the cases.^28^ TPS = (number of PD-L1-positive tumor cells)/(total number of PD-L1 positive + PD-L1-negative tumor cells).

### Outcomes and Statistical Analyses

The primary clinical endpoint was OS from start of second-line ICI monotherapy until death or loss of follow-up. To account for delayed entry into the real-world clinico-genomic cohort, risk set adjustment was performed to adjust for left truncation bias. The Kaplan-Meier method along with the log-rank test was used to estimate differences between outcome estimates. Categorical variables were compared using the 2-sided Fisher’s exact test, while the 2-sided Wilcoxon rank sum test was used to compare continuous variables. All analyses were performed using the R software^29^ version 4.0.3.

## Results

### Patient Characteristics

Overall, 621 *EGFR*- and *ALK*-wild-type non-squamous patients with NSCLC treated with second-line ICI monotherapy that fit the predefined inclusion criteria were identified. Median (interquartile range) follow-up time was 10.9 (3.7-23.4) months and as of the CGDB data cutoff date, 73.3% had died. Among the 621 patients, majority were female (53.6%), self-reported race as White (73.2%), were stage IV at initial diagnosis (64.1%), 18.7% had an ECOG status over 2 at initiation of second-line ICI monotherapy and had a history of smoking (88.4%, Table 1). 59.1% and 33.5% of the patients had received either platinum-based chemotherapy or anti-VEGF combination therapy respectively, in the first-line setting (Table 1).

**Table 1.** Demographics and clinical features of the real-world clinico-genomic cohort.

Characteristic	Patients (%; *N* = 621)
Age at initiation of second-line ICI, years, median [IQR]	69.0 [61.0-75.0]
Sex	
Male	46.4
Female	53.6
Race	
Asian	1.3
African American	6.8
White	73.2
Other	11.4
Unknown	7.3
Practice type	
Academic	3.4
Community	96.6
Tumor stage at initial diagnosis	
Stage I	11.4
Stage II	5.2
Stage III	18.2
Stage IV	64.1
Unknown	1.1
Smoking status	
History of smoking	88.4
No history of smoking	11.6
ECOG status at initiation of second-line ICI	
0	19.8
1	43.6
2	14.8
3+	3.9
Missing	17.9
First-line therapy received	
Anti-VEGF chemotherapy combination	33.5
Clinical study drugs	1.6
EGFR tyrosine kinase inhibitors	1.4
Platinum-based chemotherapy	59.1
Single agent chemotherapy	3.7
Other	0.7

### Biomarker Characteristics

Twenty percent patients (124/621) were assessed for PD-L1 IHC expression. Among them, 41.1%, 30.6%, and 28.2% patients had a PD-L1 TPS score greater than or equal to 50%, between 1% and 49% and less than 1%, respectively. The median (and inter-quartile range) TMB of the CGDB cohort (*N* = 621) was 8.8 (3.5-14.8) muts/Mb, while 45.4% of patients had a TMB greater than or equal to 10 muts/Mb. 0.5% of the cohort had MSI status of high.

### Association of CD274 Copy Number with Response to ICI Blockade

Across the overall cohort, 1.4%, 2.4%, 4.7%, and 15.0% patients had a *CD274* copy number greater than or equal to specimen ploidy +4, greater than or equal to specimen ploidy +3, greater than or equal to specimen ploidy +2, greater than or equal to specimen ploidy +1, respectively, while 36.9% patients had a *CD274* CN equal to specimen ploidy. Among patients with a *CD274* loss, 48.1%, 11.8%, and 1.1% had a *CD274* copy number lesser than or equal to specimen ploidy −1, lesser than or equal to specimen ploidy −2 and lesser than or equal to specimen ploidy −3, respectively. To examine the association of *CD274* copy number (CN) to ICI blockade, we studied the OS of patients from the start of second-line ICI monotherapy, stratified by their *CD274* CN relative to specimen ploidy, at various *CD274* CN thresholds.

**Figure 1.**
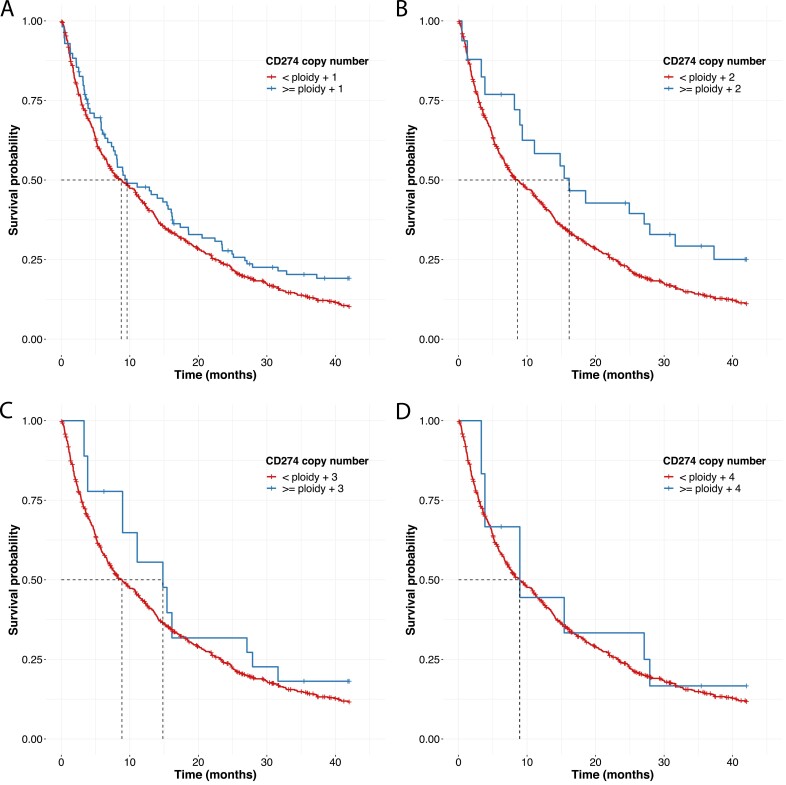
*CD274* copy number gain as a positive predictor of OS in ICI-treated non-squamous NSCLC. Overall survival (OS) of patients from start of second-line ICI monotherapy, as stratified by their *CD274* copy number (CN) relative to specimen ploidy (A) median OS (mOS) of patients with a *CD274* CN ≥specimen ploidy +1 (*N* = 93) was 9.6 [7.6-16.2] months and patients with a *CD274* CN <specimen ploidy +1 (*N* = 528) had a mOS = 8.8 [6.9-11.2] months. Hazard ratio (HR) for the ≥specimen ploidy +1 group = 0.8 [0.6-1.0], *P* = 0.09. (B) mOS of patients with a *CD274* CN ≥specimen ploidy +2 (*N* = 29) was 16.1 [8.9-37.3] months and patients with a*CD274* CN<specimen ploidy +2 (*N* = 592) had a mOS = 8.6 [7.1-10.9] months. Hazard ratio (HR) for the ≥specimen ploidy +2 group = 0.6 [0.4-1.0], *P* = .05. (C) mOS of patients with a *CD274* CN ≥specimen ploidy +3 (*N* = 15) was 14.8 [8.9-NA] months and patients with a *CD274* CN <specimen ploidy +3 (*N* = 606) had a mOS = 8.8 [7.3-11] months. Hazard ratio (HR) for the ≥specimen ploidy +3 group = 0.8 [0.4-1.5], *P* = .5 (D) mOS of patients with a *CD274* CN ≥specimen ploidy +4 (*N* = 9) was 8.94 [3.8-NA] months and patients with a *CD274* CN<specimen ploidy +4 (*N* = 612) had a mOS = 8.9 [7.3-11.2] months. Hazard ratio (HR) for the ≥specimen ploidy +4 group = 0.8 [0.4-1.9], *P* = .7.

When assessing the effect of *CD274* CN gain as a positive predictor of OS to ICI monotherapy, we identified that at a *CD274* CN threshold of greater than or equal to specimen ploidy +1, the gain group (*N* = 93) had a higher median OS (mOS, 95% confidence interval) of 9.6 [7.6-16.2]) months when compared with the rest (*N* = 538, mOS = 8.8[6.9-11.2], *P* = .09; Fig. 1A), at a *CD274* CN threshold of greater than or equal to specimen ploidy +2, the gain group (*N* = 29) had significantly higher mOS of 16.1 [8.9-37.3]) months when compared with the rest (*N* = 592, mOS = 8.6 [7.1-10.9] months, *P* = .05; Fig. 1B), at a *CD274* CN threshold of greater than or equal to specimen ploidy +3, the gain group (*N* = 15) had higher mOS of 14.8 [8.9-NA]) months when compared to the rest (*N* = 606, mOS = 8.8[7.3-11] months, *P* = .5; Fig. 1C), while at a *CD274* CN threshold of greater than or equal to specimen ploidy +4, the gain group (*N* = 9) had comparable mOS of 8.94 [3.8-NA]) months when compared with the rest (*N* = 612, mOS = 8.9 [7.3-11.2] months, P = .7; Fig. 1D). As the *CD274* copy number threshold was increased from at least specimen ploidy +1 to at least specimen ploidy + 4, the 1-year OS rate amongst the patients with *CD27*4 gains was observed to be 61.1%, 73.3%, 75%, and 66.7%, respectively.

Given the significantly higher survival at a *CD274* CN threshold of greater than or equal to specimen ploidy +2, we specifically examined the cohort using the ploidy +2 cutoff, and here we observed that there were no significant differences in the demographics and clinical characteristics of the gain group (*CD274* CN threshold greater than or equal to specimen ploidy +2) vs the rest of the patients, (Supplementary Table S1) but among the well-studied ICI biomarkers of PD-L1, TMB and MSI, TMB-High (at a threshold of 10 muts/Mb), TMB-High was significantly enriched in the gain group (Table 2). Of note, although PD-L1 protein expression data were only available for a subset of cases, *CD274* CN changes were overall correlated with PD-L1 protein expression (Supplementary Table S2). However, they were not entirely concordant and cases with *CD274* CN gain with no PD-L1 protein expression, and *CD274* CN loss with PD-L1 protein expression existed in this cohort.

**Table 2.** Comparison of ICI therapy associated biomarkers between the CD274 CN ≥specimen ploidy +2 and the *CD274* CN<specimen ploidy +2 cohorts.

Biomarker	<Ploidy +2; *N* = 592 (% (N))	≥Ploidy +2; *N* = 29 (% (N))	*P*-value
PD-L1 (TPS)			
< 1%	8.4 (50)	3.4 (1)	0.5
1%-49%	5.7 (34)	13.8 (4)	0.1
≥50%	5.6 (33)	6.9 (2)	0.7
Unknown	80.3 (475)	75.9 (22)	0.6
TMB (≥10 mutations/Mb)	44.2 (262)	69 (20)	0.01
MSI-High	0.5 (3)	0 (0)	1

*P*-values were estimated using the 2-sided Fisher’s exact test.

**Figure 2.**
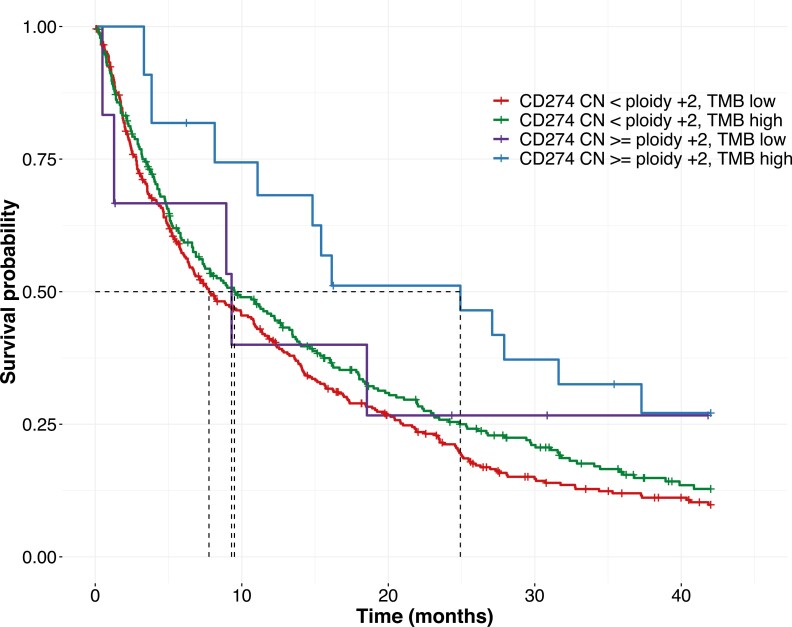
OS in ICI-treated non-squamous NSCLC as stratified by TMB (at a threshold of 10 muts/Mb) and *CD274* CN group (at a copy number gain threshold of 2). mOS of patients with *CD274* CN <ploidy +2 and TMB low (*N* = 330) was 7.7 [6.3-10.9] months, mOS of patients with *CD274* CN <ploidy +2 and TMB high (*N* = 262) was 9.5 [7.1-13.2] months, mOS of patients with *CD274* CN ≥ploidy +2 and TMB low (*N* = 9) was 9.3 [1.3-NA] months and mOS of patients with *CD274* CN ≥ploidy +2 and TMB high (*N* = 20) was 24.9 [11.1-NA] months, *P* = .04.

At a *CD274* copy number gain threshold of 2, when the OS from start of second-line ICI monotherapy was stratified by TMB-High, an additive pattern emerged. mOS of patients with *CD274* CN less than ploidy +2 and TMB low (*N* = 330) was the lowest at 7.7 [6.3-10.9] months, mOS of patients with *CD274* CN less than ploidy +2 and TMB-High (*N* = 262) was comparable with that of patients with *CD274* CN greater than or equal to ploidy +2 and TMB low (*N* = 9) at 9.5 [7.1-13.2] months and 9.3 [1.3-NA] months, respectively, while mOS of patients with *CD274* CN greater than or equal to ploidy +2 and TMB-High (*N* = 20) was the highest at 24.9 [11.1-NA] months, *P* = .04 (Fig. 2). As an exploratory analysis, we included the PD-L1 status where available in these different subgroups defined by *CD274* CN and TMB (Supplementary Table 3), although the number of cases with available PD-L1 status is small to make any conclusions.

**Figure 3.**
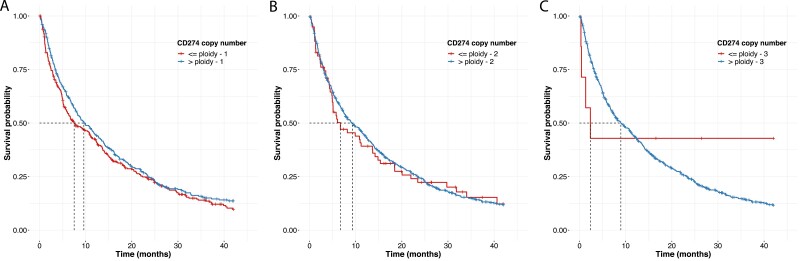
*CD274* copy number loss as a negative predictor of OS in ICI-treated non-squamous NSCLC. Overall survival (OS) of patients from start of second-line ICI monotherapy, as stratified by their *CD274* copy number (CN) relative to specimen ploidy (A) Median OS (mOS) of patients with a *CD274* CN >specimen ploidy −1 (*N* = 322) was 9.6 [7.9-12.8] months and patients with a *CD274* CN ≤specimen ploidy −1 (*N* = 299) had a mOS = 7.5 [5.9-11.3] months. Hazard ratio (HR) for the >specimen ploidy −1 group = 0.9 [0.7-1.1], *P* = .3. (B) Median OS (mOS) of patients with a *CD274* CN >specimen ploidy −2 (*N* = 548) was 9.3 [7.5-11.5] months and patients with a *CD274* CN ≤specimen ploidy −2 (*N* = 73) had a mOS = 6.7 [4.9-14.2] months. Hazard ratio (HR) for the >specimen ploidy −2 group = 0.96 [0.7-1.3], *P* = .8. (C) Median OS (mOS) of patients with a *CD274* CN >specimen ploidy −3 (*N* = 614) was 8.9 [7.4-11.2] months and patients with a *CD274* CN ≤specimen ploidy −3 (*N* = 7) had a mOS = 2.3 [0.4-NA] months. Hazard ratio (HR) for the >specimen ploidy −3 group = 1.3 [0.5-3.5], *P* = .6.

We also observed that *CD274* loss defined as a *CD274* CN lesser than or equal to specimen ploidy −1 (*N* = 299) trended toward lower mOS (mOS = 7.5 [5.9-11.3] months), when compared with the rest of the cohort (*N* = 322; mOS = 9.6 [7.9-12.8] months, *P* = .3; Fig. 3A). When the *CD274* loss threshold was lowered to a *CD274* CN lesser than or equal to specimen ploidy −2, the mOS for the loss group (*N* = 73) was 6.7 [4.9-14.2] months when compared with rest of the cohort (*N* = 548, mOS = 9.3 [7.5-11.5] months, *P* = .8; Fig. 3B) and at a *CD274* CN lesser than or equal to specimen ploidy −3, the mOS for the loss group (*N* = 7) dropped further to 2.3 [0.4-NA] months when compared with rest of the cohort (*N* = 614, mOS = 8.9 [7.4-11.2] months, *P* = .6; Fig. 3C); however, these association were not statistically significant.

## Discussion

While the importance of *CD274* gains and losses as biomarkers of response to ICI has been increasingly emphasized, no data is available on the corresponding clinically relevant and optimal *CD274* copy number thresholds. In this retrospective clinical study utilizing a large clinico-genomic database, we describe the association of ICI response with *CD274* copy number gains and losses, at different copy number thresholds, in 621 non-squamous patients with NSCLC. Specifically, we showed that *CD274* copy number gain of ploidy +2 (CN ≥ 4 in diploid tumor samples) is an optimal cutoff to predict positive response to ICI in non-squamous NSCLC. Inoue et al^20^ demonstrated in a small cohort of patients similar trends using a different assay (FISH) to define PD-L1 amplification (defined as a PD-L1 to CEP9 ratio of at least 2.0; equivalent to CN ≥ 4 in diploid tumor samples). We observed that patients with at least 4 copies of the gene had a significantly higher mOS than the rest of the cohort, but the 1-year OS rate was 73.3% compared with 100% as seen in Inoue et al, likely due to their small cohort sizes. In addition, *CD274* loss has been associated with shorter OS to ICI blockade in non-squamous NSCLC as evaluated in Lamberti et al^19^ Similarly, in our study, the *CD274* loss cohort trended toward a lower mOS when compared with the rest. Thus, these results demonstrate the positive and negative predictive value of *CD274* CN changes.

Prospective clinical trials such as the phase II trial studying the efficacy of Nivolumab and Ipilimumab in patients with rare cancers (NCT02834013) are currently enrolling patients with *CD274* amplifications, defined as at least 6 copies of *CD274* detected through CGP. This on-going trial further emphasizes the importance to define and evaluate the clinical relevance of *CD274* copy number gain thresholds used to enroll patients onto ICI-based clinical trials. In this manner, more patients can potentially be accrued and could benefit from such clinical studies. In addition, as previously described,^21^ higher rates of *CD274* gains have also been reported in a variety of tumors featuring squamous cell histology and hence it is important to identify disease specific clinically relevant *CD274* copy number gain thresholds to predict ICI response.

The current study also identifies an additive effect of *CD274* CN gain (at a threshold of at least 4 copies) and TMB on response to ICI inhibitors. The *CD274* CN low and TMB low cohort had the lowest mOS at 7.7 months and the *CD274* CN high and TMB high cohort had the highest mOS at 24.9 months, while the 2 mixed groups had a comparable mOS of approximately 9.5 months, right in between that of the 2 other cohorts. This parallels the independent and complimentary nature of PD-L1 IHC and TMB seen across multiple tumor types, including non-squamous NSCLC.^30^ Interestingly, the gain in mOS between the TMB high and TMB low groups, was much higher in the *CD274* CN high cohort (15.6 months) compared with that in the *CD274* CN low group (1.8 months). We hypothesize that the tendency of immune evasion and hence response to ICI blockade is higher in the *CD274* CN high group, specifically in the presence of a high neoantigen burden manifested in the TMB high cohort. Thus, further studies exploring the efficacy of chemotherapy, chemoimmunotherapy and immunotherapy across these 4 cohorts appears warranted and has the potential to add precision in the treatment of clinically advanced NSCLC patients.

This study has several limitations. Firstly, interpretability of the survival outcomes in the cohort of patients with a *CD274* CN of at least ploidy +4 are limited because of the small cohort size. Second, since PD-L1 IHC data were not available for most of the cases, a head-to-head comparison on the predictive power of PD-L1 IHC vs *CD274* CN gain could not be undertaken and should be considered in future studies to determine whether PD-L1 IHC or *CD274* CN is a more predictive biomarker for ICI. It is important to note that in our previous study,^21^ while *CD274* CN gains with at least ploidy +2 was positively correlated with PD-L1 IHC in NSCLC, there was a subset of PD-L1-positive patients that were negative for *CD274* CN gain and a subset of PD-L1-negative patients that were positive for *CD274* gain at a threshold of at least ploidy + 2, indicating that *CD274* CN positivity could be an independent predictive biomarker of ICPI response.

## Conclusions

In this study, the survival outcomes with ICI monotherapy in non-squamous NSCLC varies with *CD274* copy number gains defined at different cutoffs. In future validation studies, *CD274* gains defined as at least 4 copies needs to be evaluated as a biomarker of ICI response in prospective large scale clinical studies.

## Supplementary Material

oyac096_suppl_Supplementary_Materials

## Data Availability

The data underlying this article are available in the article and in its online supplementary material.
