# Supplementary material for: Association of CD274 (PD-L1) Copy Number Changes with Immune Checkpoint Inhibitor Clinical Benefit in Non-Squamous Non-Small Cell Lung Cancer
Source: Oncologist. 2022 May 22;27(9):732–9. doi: 10.1093/oncolo/oyac096 (PMC9438920; doi:10.1093/oncolo/oyac096)
Supplement: oyac096_suppl_Supplementary_Materials [file oyac096_suppl_supplementary_materials.docx]

**Supplement**

**Supplementary Table 1**: Comparison of demographic and clinical characteristics between patients with a *CD274* CN $\geq$specimen ploidy +2 and patients with a *CD274* CN$<$specimen ploidy +2

| **Characteristic** | | **< Ploidy +2 (%; N=592)** | | **>= Ploidy +2 (%; N=29)** | **P Value** |
| --- | --- | --- | --- | --- | --- |
| *Age at initiation of second line ICI, median (IQR; years)* |  | | 68.0 [62.0-74.0] | 69.0[61.0-75.0] | 0.9 |
| *Sex* | Female | | 53.9 | 48.3 | 0.6 |
| *Race* | Asian | | 1.4 | 0 | 1 |
|  | African American | | 6.8 | 6.9 | 0.7 |
|  | Other | | 11.3 | 13.8 | 0.6 |
|  | White | | 73.6 | 65.5 | 0.4 |
|  | Unknown | | 6.9 | 13.8 | 0.2 |
| *Practice type* | Community | | 96.8 | 93.1 | 0.3 |
| *Tumor stage at initial diagnosis* | Stage I | | 11.3 | 13.8 | 0.6 |
|  | Stage II | | 5.2 | 3.4 | 1 |
|  | Stage III | | 17.6 | 31 | 0.1 |
|  | Stage IV | | 64.7 | 51.7 | 0.2 |
|  | Unknown | | 1.2 | 0 | 1 |
| *Smoking status* | History of smoking | | 88.7 | 82.8 | 0.4 |
| *ECOG status at initiation of second line ICI* | 0 | | 19.4 | 27.6 | 0.3 |
|  | 1 | | 43.8 | 41.4 | 0.9 |
|  | 2 | | 15.2 | 6.9 | 0.3 |
|  | 3+ | | 3.7 | 6.9 | 0.3 |
|  | Missing | | 17.9 | 17.2 | 1 |
| *First line therapy received* | Anti-VEGF chemotherapy combination | | 34.1 | 20.7 | 0.2 |
|  | Clinical study drugs | | 1.7 | 0 | 1 |
|  | EGFR tyrosine kinase inhibitors | | 1.5 | 0 | 1 |
|  | Platinum-based chemotherapy | | 58.4 | 72.4 | 0.2 |
|  | Single agent chemotherapy | | 3.7 | 3.4 | 1 |
|  | Other | | 0.5 | 3.4 | 0.2 |

**Supplementary Table 2:** Distribution of PD-L1 IHC status across *CD274* CN gain and loss thresholds. A total of 124 (out of 621) patients had data on PD-L1 IHC status (TPS)

| *CD274* CN | **N** | **< 1%** | **1%-49%** | **>= 50%** |
| --- | --- | --- | --- | --- |
| ≥specimen ploidy +1 | 12 | 16.6% | 41.7% | 41.7% |
| ≥specimen ploidy +2 | 7 | 14.3% | 57.1% | 28.6% |
| ≥specimen ploidy +3 | 4 | 0% | 50.0% | 50.0% |
| ≥specimen ploidy +4 | 3 | 0% | 66.7% | 33.3% |
| equal to specimen ploidy | 43 | 41.9% | 32.5% | 25.6% |
| ≤specimen ploidy -1 | 69 | 55.1% | 27.5% | 17.4% |
| ≤specimen ploidy -2 | 20 | 40.0% | 30.0% | 30.0% |
| ≤specimen ploidy -3 | 3 | 100% | 0% | 0% |

**Supplementary Table 3:** Distribution of PD-L1 IHC status across TMB (at a threshold of 10 muts/Mb) and *CD274* CN group (at a copy number gain threshold of 2). A total of 124 (out of 621) patients had data on PD-L1 IHC status (TPS)

| **Biomarker Status** | **N** | **< 1%** | **1%-49%** | **>= 50%** |
| --- | --- | --- | --- | --- |
| *CD274* CN <specimen ploidy + 2 and TMB low | 61 | 39.3% | 31.2% | 29.5% |
| *CD274* CN <specimen ploidy + 2 and TMB high | 56 | 46.4% | 26.8% | 26.8% |
| *CD274* CN ≥specimen ploidy + 2 and TMB low | 5 | 20.0% | 60.0% | 20.0% |
| *CD274* CN ≥specimen ploidy + 2 and TMB high | 2 | 0% | 50.0% | 50.0% |
